# Viability Study of Serra da Estrela Dog Wool to Produce Green Composites

**DOI:** 10.3390/polym16050718

**Published:** 2024-03-06

**Authors:** Alexandra Soledade Gomes, Paulo Torrão Fiadeiro, André Costa Vieira, Joana Costa Vieira

**Affiliations:** 1 Fiber Materials and Environmental Technologies Research Unit (FibEnTech-UBI), Universidade da Beira Interior, Rua Marquês D’Ávila e Bolama, 6201-001 Covilhã, Portugal; alexandra.soledade.gomes@ubi.pt (A.S.G.); fiadeiro@ubi.pt (P.T.F.); 2 Center for Mechanical and Aerospace Science and Technologies (C-MAST-UBI), Universidade da Beira Interior, Rua Marquês D’Ávila e Bolama, 6201-001 Covilhã, Portugal; andre.costa.vieira@ubi.pt

**Keywords:** green composites, animal fibers, dog wool fiber, green epoxy resin

## Abstract

The environmental emergency has alerted consumers and industries to choose products derived from renewable sources over petroleum derivatives. Natural fibers of plant origin for reinforcing composite materials dominate the field of research aiming to replace synthetic fibers. The field of application of green dog wool composite materials needs to be reinforced and proven, as the industry is looking for more sustainable solutions and on the other hand this type of raw material (pet grooming waste) tends to grow. Hence, in the present work, the feasibility of applying natural fibers of dog origin (mainly composed by keratin) in green composites was studied. The green composites were developed using chemically treated dog wool of the breed *Serra da Estrela* (with NaOH and PVA) as reinforcement and a green epoxy resin as a matrix. The chemical treatments aimed to improve adhesion between fibers and matrix. The fibers’ composition was determined using X-ray Diffraction (X-RD). Their morphology was determined using a scanning electron microscope (SEM). The wettability of the fiber was also evaluated qualitatively by analyzing drops of resin placed on the fibers treated with the different treatments. The mechanical properties of the composites were also studied through mechanical tensile, flexural, and relaxation tests. Overall, the best results were obtained for the dog wool fibers without treatment. The tensile and flexural strength of this biocomposite were 11 MPa and 26.8 MPa, respectively, while the tensile and flexural elastic modulus were 555 MPa and 1100 MPa, respectively. It was also possible to verify that the PVA treatment caused degradation of the fiber, resulting in a decrease in mechanical tensile strength of approximately 42.7%, 59.7% in flexural strength and approximately 59% of the stress after 120 min of relaxation when compared to fiber made from untreated dog wool. On the other hand, the NaOH treatment worked as a fiber wash process, removing waxes and fats naturally present on the fiber surface.

## 1. Introduction

The concept of sustainable chemistry and the policy of banning single-use products, together with the consumerism of populations over the years, has put pressure on industries and new technologies to replace raw materials derived from petroleum with renewable resources. Due to the limited availability and high cost of fossil resources, as well as the problems associated with waste landfills and growing environmental concerns, the demand for the use of more ecological, sustainable, and environmentally responsible materials has been increasing year after year [1,2,3].

In this way, biocomposites serve these purposes, as they present attractive characteristics such as: biodegradability, reduced costs, and wide availability and/or lower energy demand in their production, making them capable of competing with traditional materials existing in the current market. This type of materials can find application in various sectors, such as automotive, marine, aerospace, structural applications, infrastructure, packaging, and electronics [1,2,3,4,5,6].

In this context, and to make up for the lack of these new materials, the need arose for chemical industries to replace their production strategy and synthesize their products from bio-based raw materials, giving birth to bio-based resins [3,7,8,9]. Bio-based resins are produced from renewable raw materials, such as sucrose, lignin, and vegetable oils, with the advantage of consuming less than 65% of the energy in production when compared to petroleum-derived resins. This fact makes them energy-efficient and safe, as they are non-toxic, and some can be biodegradable. Furthermore, as they are produced from bio-based raw materials, they can be considered renewable and recyclable. Bio-based phenolic resins, bio-based epoxy, bio-based polyurethane, cellulose acetate, and biopolyesters are examples of the most popular bio-based resins. However, it must be considered that due to the complex structures of biomass resources, it is not practical or efficient to use them directly to synthesize bio-based thermosetting resins. Therefore, biomass needs to be transformed into intermediate molecules with useful or simpler functional groups that facilitate the chemical reaction to synthesize the bio-based resin. Table 1 summarizes the bio-based elements that can give rise to thermosetting resins and other reaction by-products [10,11].

Green epoxy resin is a bio-based product, which can be synthesized using different resources such as lignin, gallic acid, cardanol, and vegetable oils. However, vegetable oils are the most suitable raw materials to synthesize this type of resin. Since they contain unsaturated double bonds, they become a better option for promoting epoxidation reactions [11]. However, despite being a bio-based resin, it does not mean that it is biodegradable. There is a trend and growing demand in the market for sustainable bio-based materials with an emphasis on their performance and strength rather than their biodegradability [7].

With the constant search for ecological materials, the replacement of synthetic fibers becomes imminent. Hence, natural fibers have emerged as a new generation of reinforcements for composites. Natural fibers have benefits over synthetic fibers, such as low cost, low density, high acoustic damping, good relative mechanical properties, low production energy consumption, abundant and renewable sources, low carbon footprint, and biodegradability, which allows them to compete with traditional synthetic fibers such as fiberglass, carbon, and aramid. In literature, the most studied natural fibers are those of vegetable origin. However, the high demand for natural fibers generates the need to research and identify new fibers that may have potential for the production of this type of materials [1,2,6,12,13,14,15]. Therefore, animal fibers are an attractive alternative for the development of sustainable composite materials. Table 2 shows some properties of natural fibers of vegetable and animal origin.

**Figure 1:**
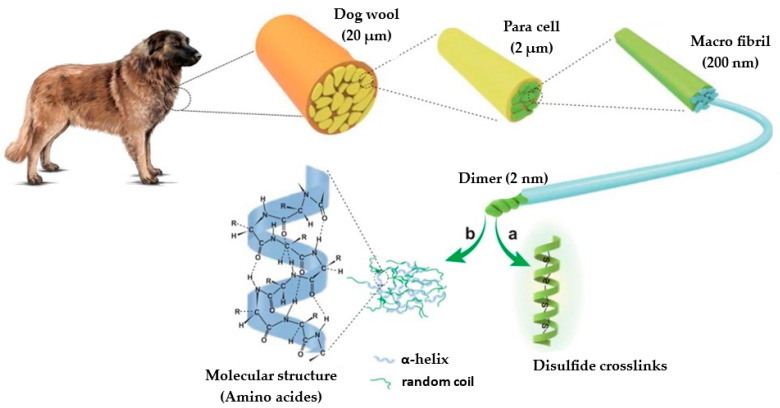
Keratin structure.

Animal fibers such as wool, feathers, hair and silk are made up of an important protein element called keratin (Figure 1). Keratin is a fibrous structural protein, considered the main constituent of wool, hair, horns, feathers, and other external coverings of mammals, reptiles, and birds. Keratin fibers have a multiscale architecture, represented in Figure 1, that makes them light and resistant. Keratin from different sources has been a research topic with a high increase in demand due to the ease with which it can be integrated into biomaterials, as well as its light weight, low cost, ecological nature, and insolubility in organic solvents. Furthermore, keratin fibers have good hydrophobic behavior and the ability to dampen sound [3,16,17].

In recent years there has been an increase in pets worldwide. With the number of pets increasing, the number of pet grooming stores has also increased. This increasing market generates a high amount of keratinous waste (animal hair) that ends up in landfills. Thus, millions of tons of keratin-based material are discarded annually around the world. Therefore, collecting this waste from pet stores and converting it into reinforcing fibers would be an innovative and sustainable way to treat this waste [3,13,18,19].

To respond to the need to develop new ecological materials and the need to identify new natural fibers, this study was developed with the aim of producing green composites with dog wool fiber waste to provide a sustainable application for this unexplored resource.

## 2. Materials and Methods

### 2.1. Materials

By definition, to produce a green composite, one needs to combine natural fibers with natural resins to create a natural composite material. In this work, the natural fiber used was an animal fiber, and the natural resin was a bio-based resin obtained from vegetable oils.

#### 2.1.1. Fibers

- Serra da Estrela dog wool

The wool from *Serra da Estrela* dog breed used to produce the green composite was obtained from brushing a long-haired *Serra da Estrela* dog, obtained during the shedding season (spring and autumn). According to Ramamoorthy et al. [13], the density of dog wool varies between 1.31–1.34 g/cm^3^, which includes the density of sheep wool 1.3 g/cm^3^. In accordance with this information, a density value of 1.3 g/cm^3^ was assumed for the calculations.

- Fiberglass

Type E-glass fiber was used to produce the traditional composite, whose properties are shown in Table 3.

#### 2.1.2. Resins 

To produce the green composite, SR GreenPoxy 56 resin was used with SD Surf Clear hardener from Sicomin (Chateauneuf les Martigues, France). The selection of the green resin used for this work was made taking into account the study carried out by Terry & Taylor [21], which concluded that among nine bio-based epoxy resins tested, the SR GreenPoxy 56 resin is one of the most promising and offers good performance for composite materials in marine applications, sports equipment, and wind energy. The traditional composite was produced with SR8100 resin and SD 3304 hardener from the Sicomin brand. The mechanical properties of the resins can be found in Table 4.

### 2.2. Methods

#### 2.2.1. Fiber Characterization

- X-ray Diffraction (X-RD)

The X-RD was carried out on a Rigaku diffractometer, model DMAX 111/C. The test was carried out with a sweep in the range between 5 and 50 degrees.

- Scanning Electron Microscope (SEM)

High-resolution images of the fibers were obtained using a HITACHI model S-3400N scanning electron microscope (SEM) (Hitachi High-Technologies Corporation, Tokyo, Japan). All analyzed samples were first coated with gold.

- Optical wettability test of *Serra da Estrela* dog wool fibers

**Figure 2:**
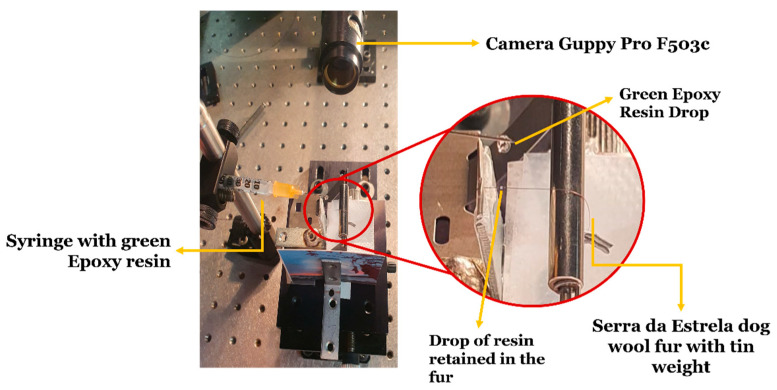
Set-up for carrying out the fiber wettability test.

The wettability of the fibers was tested using a Guppy Pro F503c model camera (Allied Vision, Stadtroda, Germany) to obtain images of the fiber-resin interaction, with a resolution of 1940 × 1292 pixels. The set-up to carry out these tests is shown in Figure 2. A tin weight with a mass of 0.15 g was placed on the dog wool fibers in order to keep the fiber stretched during the test. The images were obtained presenting a region of interest (ROI) of 586 × 1292 pixels, where each pixel has a size of 5.74 µm.

**Figure 3:**
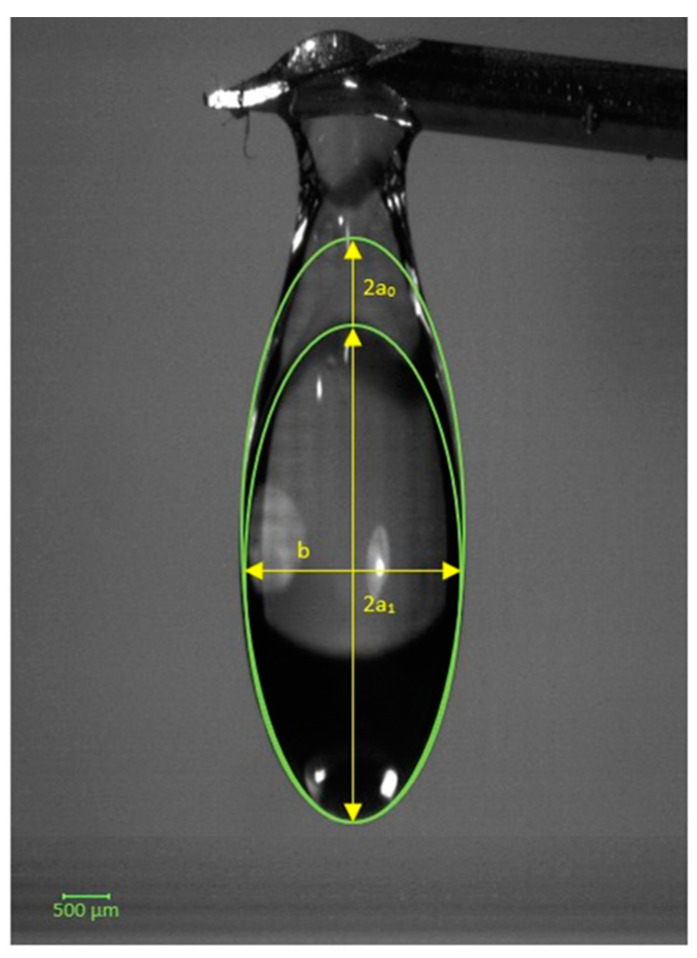
Drop of resin with demonstration of the largest radius and smallest radius.

The drop of resin has a volume of 18.53 µL. The volume of the resin drop was calculated using the volume of an ellipsoid of revolution according to Equation (1);

(1) $$V=\frac{4}{3}\pi abc\;$$

where V corresponds to the volume of the drop, a is the radius in millimeters (determined from the average between the largest radius (2a_0_) and the smallest radius (2a_1_)), b corresponds to the horizontal diameter in millimeters, and c the diameter perpendicular to the plane, also in millimeters. As it is a cigar-shaped ellipsoid of revolution, it is assumed that b=c as shown in Figure 3.

A drop of resin was deposited on a dog wool sample filament and the interaction between them was registered optically. This process was repeated for all the dog wool sample filaments under study. The wettability was qualitatively evaluated using a MATLAB^®^ R2023a application for all the registered images.

#### 2.2.2. Chemical Treatments of Serra da Estrela Dog Wool Fibers

The green composites were produced with dog wool as collected, without any treatment, and dog wool that was chemically treated. Two chemical treatments were carried out to compare which of the three types of dog wool (wool without treatment, wool with PVA treatment, and wool with NaOH treatment) performs best when embedded into the composite matrix. Thus, the samples are named according to the scheme: M_x_X_y_, where M means matrix and whose index x takes the designation of E_c_ (conventional epoxy) or E_g_ (green epoxy) and where X means reinforcement and whose index y takes the designation of F_g_ (glass fiber), F_wot_ (fiber wool without treatment), F_PVA_ (fiber wool with PVA treatment) or F_NaOH_ (fiber wool with NaOH treatment). According to Conzatti et al. [22], the PVA treatment was used on sheep’s wool. Hence, due to its similarities with dog wool from *Serra da Estrela*, it was decided to use this same treatment. On the other hand, Claudivan da Silva et al. [23] used the NaOH treatment applied to dog fur. The procedures adopted for these two treatments were adaptations of the descriptions found in the literature.

- Treatment with Polyvinyl Alcohol (PVA)

A solid-liquid extraction was carried out with a 200 mL volume of acetone for 2 h. After extraction, the dog wool fibers were washed, and the excess water was removed from the fibers through a vacuum filtration process. Then, the fibers were dried in an oven at 105 °C for 24 h. Subsequently, the fibers were soaked in a PVA solution containing 3% by weight of PVA. Finally, the fibers were dried in an oven at 60 °C for 24 h.

- Treatment with Sodium Hydroxide (NaOH)

The dog wool fibers were washed with a 0.05 M NaOH solution. The solution was changed every 30 minutes three times at 40 °C, to ensure a better washing of the fibers. After washing, the excess solution was removed using vacuum filtration, and the fibers were dried in an oven at 60 °C for 24 h.

#### 2.2.3. Production of Composite Plates

**Figure 4:**
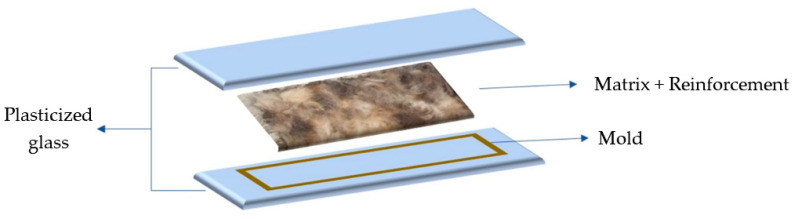
Composite plate production scheme.

The composite laminate was produced according to the scheme shown in Figure 4. The mold measures 29 × 17 × 0.1 cm. All measured weights, both the resin and the fibers used to produce the composite laminates, were initially determined to comply with the dimensions of the mold and the same volume fractions. However, after production it was found that the thickness of the dog wool sheets was greater than 0.1 cm, which in the end resulted in a different volume fraction of the laminate. In this way, the fiberglass laminate had a volume fraction of 72% resin and 28% fiberglass, while the laminates with dog wool had a volume fraction of 35% resin and 65% fiber. The composite laminates were placed under vacuum for 4 h, and curing was carried out at room temperature for 24 h. The post-curing was carried out in an oven at 40 °C for 24 h.

**Figure 5:**
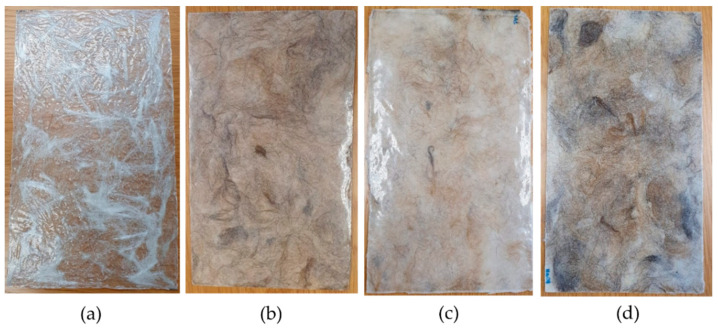
Composite laminates produced—(**a**) Fiberglass composite; (**b**) Dog wool fibers without treatment composite; (**c**) Dog wool fibers with PVA treatment composite; (**d**) Dog wool fibers with NaOH treatment composite.

The composite laminates obtained are shown in Figure 5. As can be seen in Figure 5, despite having used the same amounts of fiber and resin to produce the composite laminates, it is possible to observe that the fiberglass laminate presents a smaller thickness (less fiber volume) compared to laminates produced with dog wool fibers.

#### 2.2.4. Mechanical Tests 

From the composite laminates produced, specimens to carry out tensile, flexural, and relaxation tests were cut on a CNC machine using a water jet. Five samples were used to perform each mechanical test.

- Tensile tests

Tensile properties were determined according to ISO 527-1 [24] and ISO 527-4 [25]. Tests were carried out on a Shimadzu universal mechanical testing machine, using a 50 kN load cell, at a displacement rate of 2 mm/min, and the distance between grips was 100 mm.

- Flexural tests

The three-point bending tests were carried out in accordance with ISO 178 [26], on a Shimadzu machine, model Autograph AGS-X (Shimadzu Corporation, Kyoto, Japan) using a 10 kN load cell to determine the bending strength of the specimens. The test was carried out at a displacement rate of 1.5 mm/min and the span distance of the different types of test pieces was carried out according to the above-mentioned standard.

The rule of mixtures enables us to calculate the limit values for the global modulus of elasticity of the composite. Starting from the isostress and isostrain conditions of a composite, where the load is parallel or transverse to the fibers, respectively, the limit values for the elastic modulus of the composite are obtained by Equations (2) and (3), respectively.

(2) $$E_{C,T}=\frac{E_{m}.E_{f}}{V_{m}.E_{f}+V_{f}.E_{m}}\;$$

where $E_{C,T}$ represents the isostress condition, $E_{m}$ and $E_{f}$ the elastic modulus of the matrix and fiber in N/mm^2^, and $V_{m}$ and $V_{f}$ represent the volume fraction of the matrix and fiber, respectively.

(3) $$E_{C,L}=E_{m}.V_{m}+E_{f}.V_{f}$$

where $E_{C,L}$ is the isostrain condition, $E_{m}$ and $E_{f}$ represent the elastic modulus of the matrix and fiber in N/mm^2^, and $V_{m}$ and $V_{f}$ represent the volume fraction of the matrix and fiber, respectively.

- Relaxation Tests

The relaxation tests were carried out on a Shimadzu machine, model Autograph AGS-X, using a 10 kN load cell according to ASTM E328 [27]. The specimens used in the test had the same dimensions as those used in the flexural tests. The load was applied at a displacement rate of 200 mm/min, and the deflection applied at mid-span for the E_g_F_wot_, E_g_F_NaOH_, and E_g_F_PVA_ composites was 2 mm, while for the E_c_F_g_ it was 1 mm.

The Kohlrausch-Williams Watts (KWW) model was used to predict the viscoelastic response, since it has been shown in the literature to be more appropriate to model stress relaxation [28,29,30,31]. According to this model, the stress evolution over time is given by Equation (4):

(4) $$\emptyset =\frac{\sigma \left(t\right)}{\sigma_{0}}=e^{{-}^{{\left(\frac{1}{\tau }\right)}^{\beta }}}$$

where ∅ is the normalized stress relaxation function, σ(t) is the stress at a given time, σ_0_ is the initial stress at t = 0, β is a dimensionless parameter known as the fractional power exponent, and τ is the KWW relaxation time [28]. 

## 3. Results

### 3.1. Fiber Characterization

#### 3.1.1. X-ray Diffraction (X-RD)

**Figure 6:**
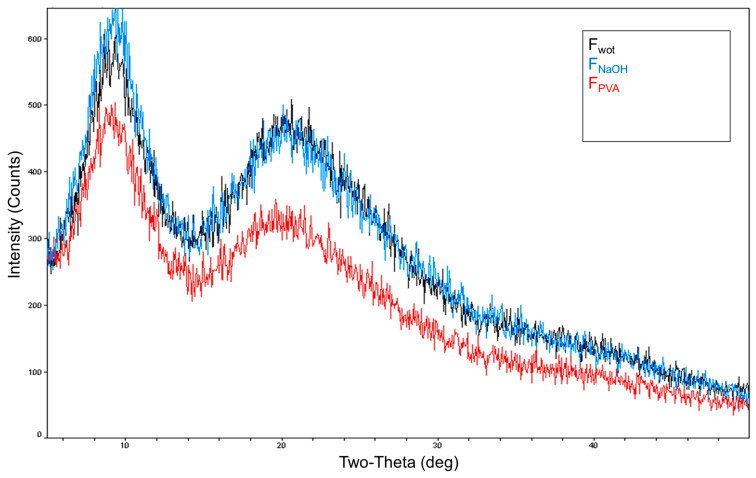
X-RD curves for *Serra da Estrela* dog wool fibers: without treatment (F_wot_—black); with PVA treatment (F_PVA_—red); with NaOH treatment (F_NaOH_—blue).

The X-RD characteristic curves obtained for *Serra da Estrela* dog wool fibers without treatment (F_wot_), with PVA treatment (F_PVA_), and with NaOH treatment (F_NaOH_), are shown in Figure 6.

#### 3.1.2. SEM

**Figure 7:**
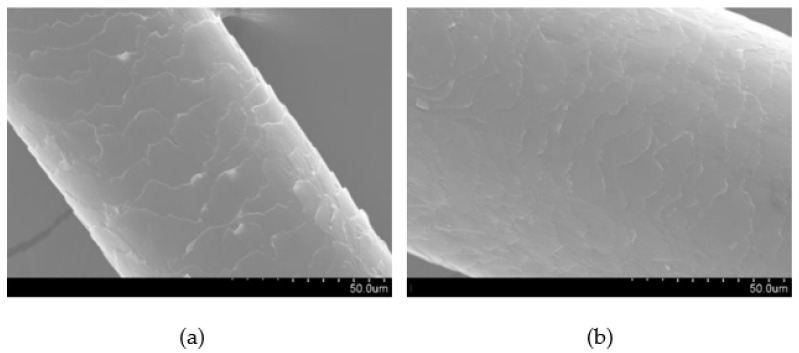
Images obtained with SEM at 1.00 K magnification: (**a**) dog wool fibers with NaOH treatment (F_NaOH_); (**b**) dog wool fibers without treatment (F_wot_).

The images obtained using an SEM for F_NaOH_ e F_wot_ are presented in Figure 7.

**Figure 8:**
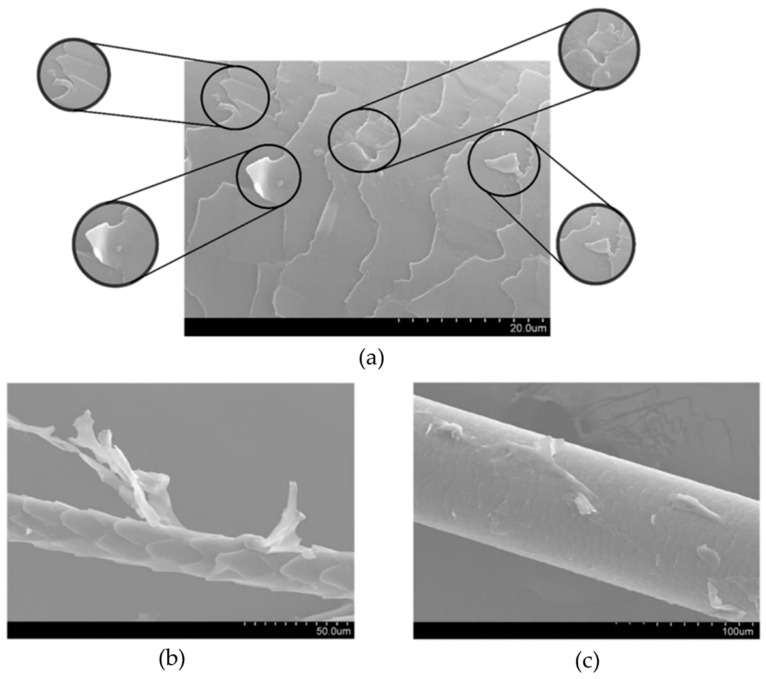
Images obtained with SEM: (**a**) dog wool fibers (F_PVA_) with PVA treatment—magnification 1.00K; (**b**) Undercoat dog wool fibers with PVA treatment—magnification 750; (**c**) Undercoat dog wool fibers with PVA treatment—magnification 500.

Figure 8 shows the images obtained for dog wool fibers (F_PVA_) and for the undercoat dog wool fibers, both with PVA treatment.

**Figure 9:**
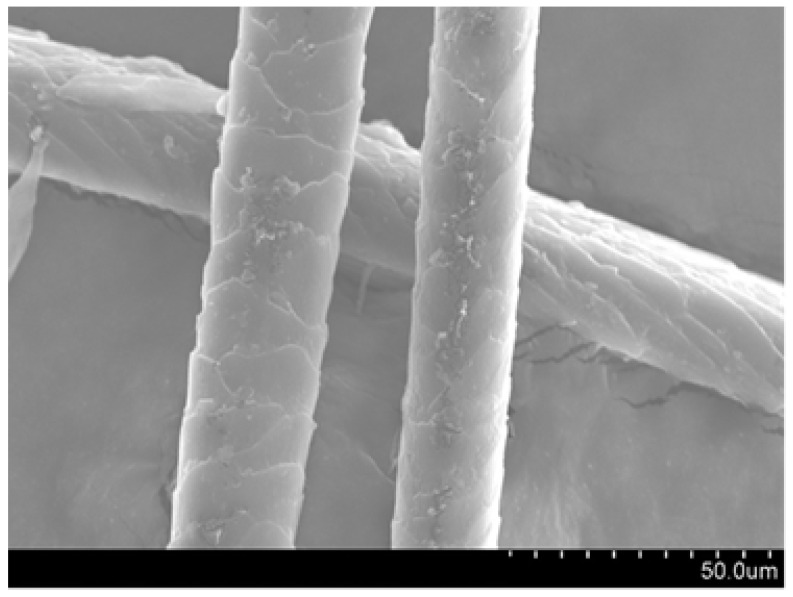
Image obtained with SEM of undercoat dog wool fibers without treatment—magnification 750.

The image of the undercoat dog wool fibers without treatment obtained using an SEM is shown in Figure 9.

#### 3.1.3. Optical Wettability Test of Serra da Estrela Dog Wool Fibers

**Figure 10:**
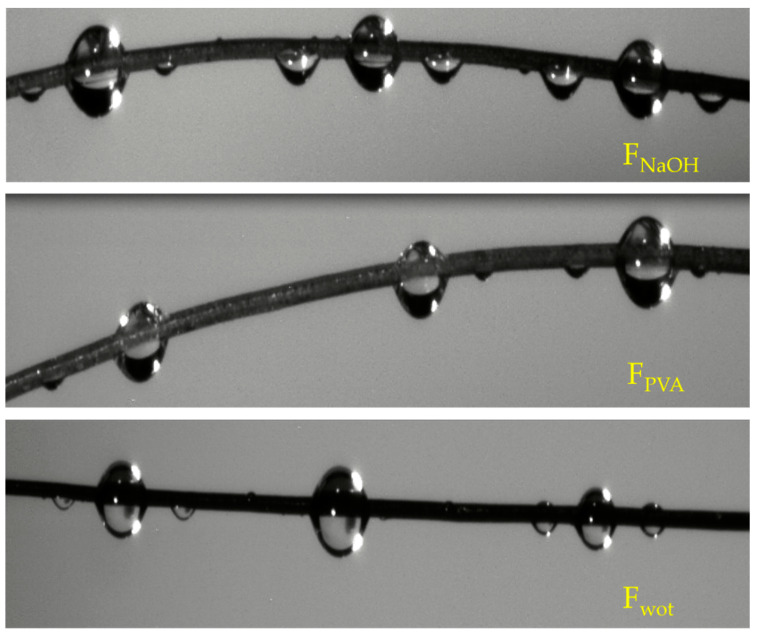
Wetting of *Serra da Estrela* dog wool fiber with green epoxy resin.

In Figure 10 it is possible to observe the behavior of a drop of green epoxy resin when in contact with the fiber.

### 3.2. Mechanical Tests

#### 3.2.1. Tensile Tests

**Figure 11:**
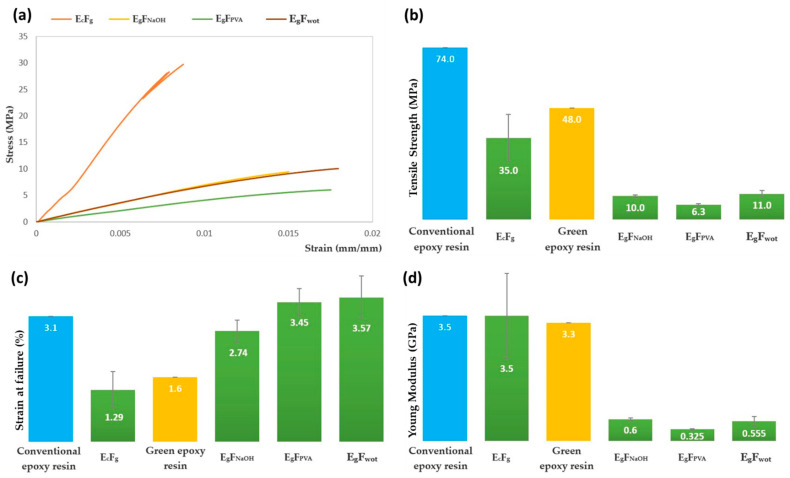
(**a**) Tensile test stress/strain graph; (**b**) Tensile strength results (MPa); (**c**) Strain at failure results (%) and (**d**) tensile Young’s Modulus results (GPa).

The graphs of the average stress versus strain from the tensile test results, the tensile strength results, the strain at failure results, and the tensile Young’s modulus values for the different resins and composite laminates are shown in Figure 11.

Table 5 presents the results of the elastic modulus obtained experimentally and those estimated using the rule of mixtures in both the isostress and isostrain conditions.

#### 3.2.2. Flexural Tests

Toughness was estimated numerically from the area under the curve of the stress/strain graph up to failure. The toughness values thus obtained are found in Table 6.

**Figure 12:**
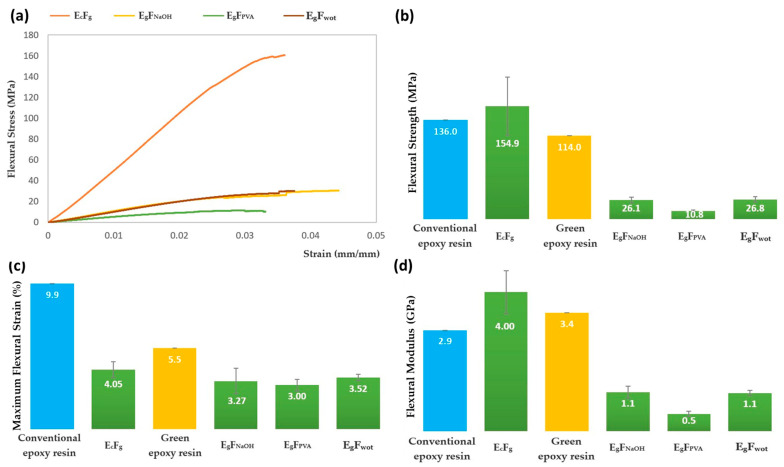
(**a**) Graph of the average stress/strain curve due to flexural loading; (**b**) flexural strength results (MPa); (**c**) maximum flexural strain results (%) and (**d**) flexural Modulus results (GPa).

The graphs of the average stress versus strain due to flexural loading results, the flexural strength results, the maximum flexural strain results, and the flexural Young’s modulus values for the different resins and composite laminates are shown in Figure 12.

#### 3.2.3. Relaxation Tests

**Figure 13:**
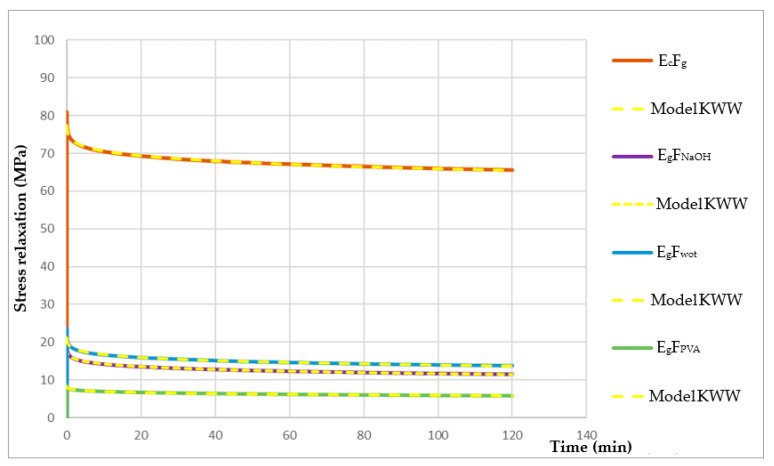
Graph of stress relaxation (MPa) along a Time axis (min) with the KWW model adjustment.

Figure 13 shows the graph of stress relaxation along a time axis.

Table 7 shows the values of the parameters obtained from the KWW model.

## 4. Discussion

### 4.1. Fiber Characterization

#### 4.1.1. X-ray Diffraction (X-RD)

As shown in XRD, dog wool is mainly composed of keratin. Keratin is a long chain, strong, and flexible protein that can be used as a reinforcement in the production of composite materials. It is commonly used in the production of biocomposites, which are materials made from a combination of natural fibers and a polymer matrix. Keratin can be easily integrated into these materials, making them more sustainable and environmentally friendly. Dog wool is suitable for processing into composite materials and an alternative source of keratin.

Analyzing Figure 6, it is possible to observe that in all wool samples two strong diffraction peaks were observed at 2θ angles, one around 9° and the other around 20°, corresponding to the α-helix and β-pleated structures of keratin, respectively [32,33,34]. The diffraction peak at the 2θ angle of around 20° appears wider due to the stacking of the β-pleated structures [34]. Interestingly, when we compare the curve obtained for *Serra da Estrela* dog wool without treatment (Figure 6 (F_wot_—black)) to those with the respective treatments (Figure 6 (F_PVA_—red) and (F_NaOH_—blue)), we found that the treatment with PVA (Figure 6 (F_PVA_—red)) showed a lower diffraction intensity in both peaks (both structures—α-helix and β-pleated), suggesting a decrease in the crystallinity of the material, while the treatment with NaOH (Figure 6 (F_NaOH_—blue)) showed a greater diffraction intensity in both peaks (both structures—α-helix and β-pleated) an effect more pronounced in the 9° peak (i.e., in the α-structure helix), suggesting greater crystallinity of the material. From the studies by Liu et al. [32] and Wu et al. [34], we can see that both keratin and sheep wool have two diffraction peaks at these same two 2θ angles (9° and 20°), with keratin having diffraction peaks with weaker intensities, indicating lower crystallinity, mainly in the first peak at 9°, i.e., fewer α-helix structures. After treatment with PVA, the solvent was recovered in a rotary evaporator, obtaining an oily residue, but not in sufficient quantity to be analyzed. Obtaining this residue may explain the difference in crystallinity between the two treatments. Since these components of the dog wool were removed, its structure was affected and consequently so was the adhesion to the matrix and the mechanical properties of the dog wool itself. 

#### 4.1.2. SEM

Observing the images obtained through the scanning electron microscope, we can see that in Figure 7 the scales present on the F_NaOH_ surface are clearer compared to F_wot_. This is because the NaOH treatment removes the waxes and fats naturally present in dog wool. It is also possible to verify that the treatment did not cause fiber degradation, which indicates that the NaOH treatment is a good fiber-washing process. 

What is seen in F_PVA_, presented in Figure 8, is that the treatment caused degradation of the fiber. Since PVA is a polymer, it polymerized randomly on the surface of the fiber, causing the irregularities visible in Figure 8b. Both PVA and keratin wool contain hydroxyl groups that can form hydrogen bonds with each other. This interaction helps the binding of PVA molecules into the keratin of the wool’s surface. On the other hand, non-polar regions of PVA interact with the non-polar regions of keratin wool through van der Waals forces. This interaction promotes the adhesion between PVA and keratin wool. These two types of interactions contribute to creating a strong bond between the polymer and the protein material. PVA can be crosslinked with keratin wool using appropriate crosslinking agents such as glutaraldehyde or epoxy compounds, as in the case of this study. This chemical crosslinking forms covalent bonds between PVA and keratin wool, leading to improved adhesion and mechanical properties. This last type of connection was not found in practice, since the PVA treatment of the fibers involves a first stage of extraction with acetone. Acetone is a strong solvent that can break down the structure of the keratin fibers, leading to a loss of integrity and potentially compromising the quality of the extracted material. The harsh nature of acetone can also cause damage to the keratin fibers, resulting in breakage or weakening of the material. It was found that degradation of the fiber actually occurred, as shown by the poor mechanical results obtained for this treatment. Consequently, the binding to PVA was not uniform throughout the fiber, as can be seen in the images presented in Figure 8.

In Figure 9, it is possible to see that the scales of the F_wot_ undercoat have different geometries and diameters, ranging from 19.3 µm to 24.9 µm.

#### 4.1.3. Optical Wettability Test of Serra da Estrela Dog Wool Fibers

The fiber wettability test was initially carried out with water, with which it was possible to notice that the fiber was unable to retain any fluid. Subsequently, the test was carried out using the green epoxy resin, where it is possible to see in Figure 9 that in F_wot_ samples the resin drops in contact with the fiber revealed a qualitative smaller contact angle. In F_NaOH_ samples, the contact angle of the resin droplet with the fiber is slightly lower, which indicates that the fiber has better resin wettability. This is because the waxes and fats present in the fiber have been removed with the NaOH treatment, thus allowing better adhesion of the resin to the fiber. On the other hand, it is possible to see that when the drop of resin is in contact with the fiber, it divides into two more drops. This is because the scales present in the wool increase its surface roughness (Figure 7). However, in F_PVA_ this phenomenon does not occur in the same way due to the degradation of the fiber by the PVA treatment (Figure 8).

### 4.2. Mechanical Tests

#### 4.2.1. Tensile Tests

Analyzing the tensile test results in Figure 11a,b, we can see that E_c_F_g_ has the highest tensile strength compared to the other composites, which would be expected. However, when analyzing the composites with dog wool fiber, we realized that the E_g_F_wot_ composite has a tensile strength very similar to E_g_F_NaOH_, meaning that the treatments carried out in this case did not improve the performance of the composites in the tensile test. Even so, the E_g_F_PVA_ performance was much lower, which confirms that there was indeed fiber degradation, as previously discussed in the fiber characteristics determined using X-RD and SEM. The fact that the performance of the E_g_F_NaOH_ composite is identical to E_g_F_wot_ also indicates that the NaOH treatment did not affect the fiber, from which it can be concluded that this treatment is nothing more than a fiber-washing process. From our analysis of the results, it was also possible to verify that the composites reinforced with dog wool fiber presented greater homogeneity than the E_c_F_g_, since the standard deviation of experimental results is much smaller. The tensile strength of conventional epoxy resin is 74 MPa. Comparing it with the E_c_F_g_ composite, one would expect to obtain a value at least equal to that of the resin. However, this was not verified due to the poor distribution of the glass fibers in the composite plate (see Figure 5a) and the low volume fraction that resulted more in a defect that introduces stress concentrations into the matrix. Turning to the green epoxy resin, it has a tensile strength of 48 MPa. As expected, since the wool volume fraction is higher than 50% and dog wool fiber is less resistant, this resulted in a decrease of the matrix tensile strength.

Regarding strain at failure (Figure 11c), we can see that E_c_F_g_ is the one with the lowest strain at failure, while the remaining composites with and without treatments present very similar values. In line with the results discussed previously for tensile strength, the composite laminates produced with green epoxy resin (having a strain at failure of 1.6%) showed a higher value of strain at failure, which is justified by the high volume of dog wool. The laminate produced with conventional epoxy resin showed a decrease in the value of the strain at failure (3.1%, according to Table 4) when compared to the E_c_F_g_ laminate. This is because the fiber is poorly distributed and results in a defect that impairs the mechanical performance of the composite.

According to Figure 11d, we can see that the Young’s modulus of E_c_F_g_ is 3.5 GPa, the same as conventional epoxy resin. This indicates that the specimen is mainly composed of resin. This is because the volumetric fraction of fiber is quite low in relation to the resin fraction used. Furthermore, the Young’s modulus of glass fiber is 73 MPa (see Table 3), which means that the result obtained should have been much higher if the fiber was correctly reinforcing the composite. In the E_g_F_wot_, E_g_F_NaOH_, E_g_F_PVA_ composites, the Young’s modulus decreases when compared to that of the Green Epoxy resin (3.3 GPa, according to Table 4). This is because the volume fraction of dog wool hair is much higher than that of the resin. According to the author Haris et al. [35], there is a linear relationship between the increase in fiber content and the increase in the elastic modulus of the composite, which validates the result obtained.

From the tensile test results obtained and comparing them with the results presented in Table 2, we can infer that dog wool fiber, despite having the same density as sheep wool, has quite inferior tensile strength and Young’s modulus.

Analyzing the experimental results of the Young’s modulus presented in Table 5, we can verify that they are not between the limits predicted by the theoretical models of the rule of mixtures, being closer to the modulus considering the isostress condition. The decrease in the experimental value, compared to the theoretical value using the rule of mixtures in the isostress condition, may be related to the presence of air bubbles present in the composite which has a volume fraction that has not been quantified.

#### 4.2.2. Flexural Tests

Analyzing the graph in Figure 12a, we can see that in flexural tests, the composites maintain the same behavior trend as in tensile tests. E_c_F_g_ has the highest flexural stress value of 154.9 MPa, while for E_g_F_NaOH_ and E_g_F_wot_ it is approximately 26 MPa. For E_g_F_PVA_, it is 10.8 MPa, which is the lowest value and much lower than that of other composites, which would be expected due to the degradation of the fiber by the PVA treatment, as discussed previously.

Analyzing the results obtained for the toughness of the composites presented in Table 6 for the different composites, it is possible to verify that the E_c_F_g_ can absorb greater energy until rupture. This would be expected since fiberglass has greater strength and stiffness compared to *Serra da Estrela* dog wool, meaning that E_c_F_g_ can absorb greater energy until rupture. Of the composites produced with *Serra da Estrela* dog wool fiber, E_g_F_NaOH_ has greater toughness, which represents a slightly better performance than E_g_F_wot_. This fact indicates that washing the fiber with NaOH effectively improved adhesion, and consequently the performance of the composite. On the other hand, E_g_F_PVA_, has a much lower toughness, and the elastic modulus is also lower, due to the degradation of the fiber caused by the treatment with PVA, as shown in Figure 8.

Comparing the flexural strength results presented in Figure 12b of the E_c_F_g_ composite with those of the conventional epoxy resin (136 MPa, see Table 4) and considering the high standard deviation of results due to the poor distribution of fibers in the composite and the high volume fraction of resin, there was no great difference between their values. Regarding the composites of E_g_F_wot_, E_g_F_NaOH_, E_g_F_PVA_, the value of flexural strength is lower compared to that of the green epoxy resin (114 MPa, see Table 4), since the volume fraction of the dog wool is much higher than that of the resin.

Analyzing the result of maximum flexural strain (Figure 12c) of the E_c_F_g_ composite and comparing it with that of conventional epoxy resin (9.9%, according to Table 4), there was a decrease in the value. Although in this composite the resin is in a greater volumetric fraction than the glass fiber, it is poorly distributed throughout the composite, compromising the resin’s mechanical flexural performance. In the composites produced with dog wool and green epoxy resin, there was also a decrease in the value of maximum flexural strain when compared to the value of the resin (5.5%, according to Table 4).

According to Figure 12d, we can see that E_c_F_g_ presents a higher value when compared to conventional epoxy resin (2.9 GPa), since the fiber has greater rigidity and consequently increases the rigidity of the composite. In the composites produced with *Serra da Estrela* dog wool, E_g_F_NaOH_ and E_g_XF_wot_ presented the same value, demonstrating once again that treatment with NaOH does not degrade the fiber. When compared to green epoxy resin, there was a decrease in the flexural rigidity modulus value due to the high volume fraction of dog wool hair, which has a lower stiffness than fiberglass, thus reducing the stiffness of the composite. The E_g_F_PVA_ presents a much lower value due to the degradation of the fiber caused by the PVA treatment.

#### 4.2.3. Relaxation Tests

Analyzing the graph in Figure 13, we can see that E_g_F_NaOH_ and E_g_F_wot_ present very similar viscoelastic behavior, which once again allows us to conclude that the NaOH treatment works as a fiber wash. Treatment with PVA supported the lowest load while E_c_F_g_ supported the highest. These results are in accordance with the flexural modulus and reinforce the results discussed above, since the behavior of the composites follows the same trend in all the mechanical tests carried out. We can also conclude that the KWW model fits the data successfully.

## 5. Conclusions

The dog wool used as a new source of natural fibers (keratin fibers) positively contributes to sustainability and a circular economy. The possibility of incorporating dog wool into biocomposites was studied in this work. Due to the expansion of the pet grooming market (expected to increase by 60% by 2033) [36], a greater availability of dog wool waste worldwide may become a business opportunity. To the authors’ knowledge, this is the first study of dog wool combined with epoxy resin to produce green composites.

In this work, it is possible to conclude that a homogeneous composite can be produced with a good distribution of dog wool, since the standard deviation obtained was low in the different tests carried out. On the other hand, in the composite produced with glass fiber, the standard deviation obtained was high, which indicates that this will not be the best method of producing composites with this type of fiber, as it was not possible to distribute the glass fiber homogeneously.

Furthermore, it was possible to verify that PVA is a very aggressive treatment for this type of fiber, once it was verified that the crystallinity of the fiber decreased (Figure 6). This indicates that there was degradation of the fiber, that is, the fiber was weakened, and the PVA treatment may have compromised its mechanical performance. It may also have compromised the adhesion of the fiber to the resin, which consequently led to the composite’s poor mechanical performance. Due to the degradation caused in the fiber by the PVA treatment, there was a decrease in mechanical tensile strength of approximately 42.7%, 59.7% to flexural strength, and approximately 59% stress after 120 min of relaxation when compared to dog wool fiber without treatment. On the other hand, the NaOH treatment worked as a wash of the fiber, removing the waxes and fats naturally present on the surface of the fiber. This enabled a very similar mechanical performance compared to dog wool without any treatment.

Composites produced with *Serra da Estrela* dog wool fiber have advantages compared to fiberglass composites, as they have lower production costs in addition to being ecological and using a residual by-product, which alleviates the pressure on landfills. Although they cannot be applied to structural applications that require particularly high mechanical strength and stiffness, they are appropriate as a composite material, for instance, car interior finishing panels (door panels, vehicle interior covers), decorative panels, partition walls, and pieces of furniture as coverings. However, for structural applications it is possible to use the *Serra da Estrela* dog wool fiber composite in the inner layer of sandwich panels, as an alternative to polymeric foams, wood, or honeycombs.

To improve the mechanical properties, it is proposed that future work study methods of spinning dog wool and subsequently weaving and knitting it to produce a biocomposite from a fabric. This more resistant structure of reinforcing fibers could also be used in other composite production technologies, such as infusion and RTM (Resin Transfer Molding). In the current fiber architecture, the progression of the resin flow front would drag the fibers, altering their homogeneous distribution. 

## Figures and Tables

**Table 1:** Natural resources for the development of bio-based products.

Resource	Related Chemicals	Functional Groups Where the Reaction to Produce the Bio-Based Products Occurs	Bio-Based Products
Carbohydrate	Itaconic acid 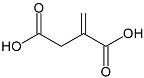	Carboxyl; c-c double bond	Epoxy resin; Polyester resin; Reactive polyester resin diluents
	Furfuryl amine 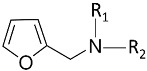	Furan	Benzoxazine; Epoxy resin
	Isosorbide 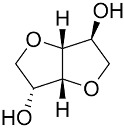	Alcohols; Diheterocycles	Epoxy resin; Polyester resin; Healing agents
Lignin	Vanillin 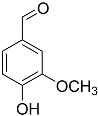	Aldehyde	Benzoxazine; Epoxy resin
	Eugenol 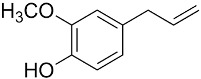	c-c double bond	Benzoxazine; Epoxy resin
	Guaiacol 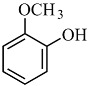	c-c double bond	Reactive polyester resin diluents
Vegetable oils	Glyceride 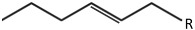	Ester; R = Unsaturated aliphatic chain	Epoxy resin; Curing agents; Polyester resin; Reactive polyester resin diluents
	Cardanol 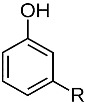	R = Unsaturated aliphatic chain	Benzoxazine; Epoxy resin

**Table 2:** Some vegetable and animal fibers’ characteristics (adapted from [16]).

Fiber	Density (g/cm^3^)	Tensile Strength (MPa)	Tensile Young’s Modulus (GPa)
Cotton	1.5–1.6	287–800	5.5–13
Linen	1.4	345–1830	27–80
Hemp	1.4	550–1110	58–70
Sheep wool	1.3	50–315	2.3–5
Silk	1.3	100–1500	5–25
Feathers	0.9	100–203	3–10

**Table 3:** Properties of E-type fiberglass (adapted from [20]).

Properties of Type E-Glass Fiber	
Tensile strength of virgin fiber (MPa)	3140
Young’s modulus (GPa)	73
Density (g/cm^3^)	2.54
Stretching (%)	4.8
Softening point temperature (°C)	850

**Table 4:** Mechanical properties of resins.

	SR GreenPoxy 56 + SD Surf Clear Hardener	SR8100 + SD 3304 Hardener
**Tensile**		
Young’s modulus (GPa)	3.3	3.5
Maximum strength (MPa)	49	74
Tensile strength (MPa)	48	74
Strain at max. load (%)	1.6	3.1
Strain at failure (%)	1.6	3.1
**Flexural**		
Young’s modulus (GPa)	3.4	3.1
Flexural strength (MPa)	114	136
Strain at max. load (%)	4.2	5.7
Strain at failure (%)	5.5	9.9

**Table 5:** Results of Young’s modulus for the different composites obtained experimentally and estimated using the isostress and isostrain conditions.

	Isostress Condition (GPa)	Isostrain Condition (GPa)	Experimentally Obtained (GPa)
E_c_F_g_	4.23	22.67	3.50
E_g_F_wot_	2.59	2.69	0.56
E_g_F_NaOH_			0.60
E_g_F_PVA_			0.33

**Table 6:** Toughness results of the different composites.

	Toughness (J/mm^3^)
E_c_F_g_	3.25
E_g_F_wot_	0.65
E_g_F_NaOH_	0.84
E_g_F_PVA_	0.24

**Table 7:** Values of the parameters obtained from the KWW model.

	KWW Model Parameters	
Composites	τ	β
E_c_F_g_	799,655	0.1773
E_g_F_wot_	3469.26	0.1829
E_g_F_NaOH_	2220.01	0.1929
E_g_F_PVA_	6442.52	0.2386

## Data Availability

Data are contained within the article.
